# A familial case of Kallmann syndrome: novel variants in ANOS1 and GNRHR genes

**DOI:** 10.20945/2359-4292-2024-0211

**Published:** 2025-09-28

**Authors:** Ana L. Piedra Pacheco, Luis F. Moya Porras, Ana B. Santos Rojo, Anthony Hong Lo, Jose E. Esquivel Vargas, Laura Ulate Oviedo

**Affiliations:** 1 Universidad de Costa Rica, Escuela de Medicina, San Jose, Costa Rica; 2 Caja Costarricense del Seguro Social, Pérez Zeledón, Costa Rica; 3 Caja Costarricense del Seguro Social, Heredia, Costa Rica

**Keywords:** Kallmann syndrome, hypogonadotropic hypogonadism, genotype-phenotype association

## Abstract

Kallmann syndrome (KS) is a rare genetic disorder characterized by hypogonadotropic hypogonadism and anosmia or hyposmia, stemming from the defective migration of GnRH and olfactory neurons during embryogenesis. This study investigated a multigenerational family with KS, identifying novel mutations in the *ANOS1* (c.78_108del, X-linked) and *GNRHR* (c.974del, autosomal recessive) genes through genetic testing. Affected males carrying the *ANOS1* mutations displayed a range of phenotypes, all of which were associated with hypogonadism and varying degrees of anosmia. Furthermore, isolated mutations in the *GNRHR* gene were linked to milder forms of hypogonadism. Individuals possessing mutations in both genes exhibited more severe phenotypes, suggesting a digenic mode of inheritance. These findings broaden the known mutational landscape of KS, illustrating how variations and combinations of mutations in multiple genes can lead to diverse symptoms and levels of severity within the same disorder. By integrating clinical and genetic data, this research enhances understanding of the intricate mechanisms underlying KS, highlighting the value of next-generation sequencing in revealing oligogenic contributions to endocrine disorders for improved diagnostics, management, and genetic counseling.

## INTRODUCTION

Kallmann syndrome (KS) is a rare genetic disorder characterized by congenital hypogonadotropic hypogonadism (CHH) and anosmia or hyposmia (^1^). It arises from defective intrauterine neuronal migration of gonadotropin-releasing hormone (GnRH) neurons from the olfactory placode to the hypothalamus (^2^). Congenital hypogonadotropic hypogonadism is defined by an impaired secretion or action of GnRH without other pituitary hormone deficiencies or structural parasellar lesions, leading to the failure of gonadal hormone secretion and gametogenesis. It may present with either anosmia, in the case of KS, or a normal sense of smell, referred to as normosmic CHH (nCHH) (^3^). KS accounts for approximately 50% of all CHH cases (^2^).

Kallmann syndrome is estimated to affect 1 in 30,000 males, whereas its prevalence in females is much lower, at 1 in 125,000, likely underestimated because affected women often experience milder symptoms (^1^). Furthermore, KS has significant genetic and phenotypic variability. Clinical features include delayed puberty, infertility, cryptorchidism, and micropenis. Alongside anosmia, KS may present with various non-reproductive characteristics such as midline defects, dental anomalies, digit abnormalities, hearing impairment, bimanual synkinesis, and renal abnormalities (^4^).

The genetic basis of KS is highly diverse, with over 50 genes associated with its development. Among these, *ANOS1*, formerly known as *KAL1*, an X-linked gene encoding anosmin 1, is one of the earliest identified and most extensively studied (^5^). This mutation accounts for 8%-20% of KS cases KS (^2^).

Another gene associated with KS and normosmic CHH is *GNRHR*, encoding the GnRH receptor, crucial for GnRH signaling in the hypothalamic-pituitary-gonadal axis. Autosomal recessive mutations in *GNRHR* typically result in isolated hypogonadotropic hypogonadism without anosmia, contributing to the phenotypic variability seen in CHH (^6^).

Despite advancements in next-generation sequencing, many KS cases remain genetically unexplained, suggesting possible undiscovered mutations or new mechanisms. Recent research highlights increasing recognition of digenic or oligogenic inheritance models, where variations in multiple genes influence the phenotype. This underscores the importance of family studies in detecting these patterns and clarifying the complex genetics of KS (^3^).

This study reports on a multigenerational family with novel mutations in *ANOS1* and *GNRHR*, contributing to the expanding mutational spectrum of KS. These findings broaden the range of mutations associated with KS and suggest a potential combined effect of mutations in these two genes on the severity of the condition.

## CASES PRESENTATION

This report summarizes the clinical records of a multigenerational family with multiple members diagnosed with KS. Genetic studies were conducted at Harvard Reproductive Sciences Center (USA) using next-generation sequencing. A total of 58 genes previously implicated in hypogonadotropic hypogonadism were analyzed.

**Figure 1:**
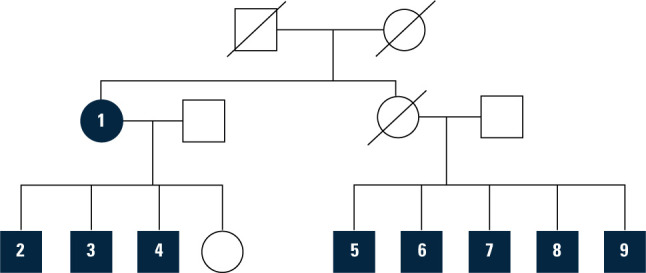
Genogram representation of the family under study Note: Own elaboration.

The first family member (herein identified as “FM” and a number) was F5, after which his relatives were assessed. Joint genetic studies revealed a frameshift mutation in the *ANOS1* gene, which is X-linked (c.78_108del, p.Pro27Serfs18), and another frameshift mutation in the *GNRHR* gene, which is inherited in an autosomal recessive manner (c.974del, p.Tyr325Phefs6). However, the members did not exhibit the same set of mutations. The affected family members’ genogram is summarized in **Figure 1**.

Among the affected members was F1, a 65-year-old female carrier of both the *ANOS1* and *GNRHR* mutations, who exhibited no symptoms of hypogonadism or anosmia.

F2, a 33-year-old male, demonstrated a range of symptoms including bilateral cryptorchidism, self-observed anosmia, a high-pitched voice, Tanner stage 2, and scrotal atrophy. Laboratory tests indicated severely suppressed gonadotropins, with Luteinizing hormone (LH) and follicle-stimulating hormone (FSH) levels at 0.21 and 0.48 mIU/mL, respectively, alongside low testosterone levels (76 ng/dL) before treatment.

F3, a 27-year-old male, presented with hypogonadism but no anosmia. His pre-treatment lab results showed low levels of LH at 0.08 mIU/mL, FSH at 0.15 mIU/mL, and testosterone at 253 ng/dL.

F4, a 26-year-old male, also displayed signs of hypogonadism along with mild self-observed anosmia. His hormonal profile included LH at 0.18 mIU/mL, FSH at 0.23 mIU/mL, and testosterone at 154 ng/dL.

F5, a 62-year-old male, experienced sensorineural hearing loss, self-observed anosmia, and bilateral cryptorchidism, with initial lab results showing low testosterone levels of 140 ng/dL and suppressed gonadotropins, including LH at 0.27 mIU/mL and FSH at 0.85 mIU/mL.

F6, a 60-year-old male, entered puberty at age 15 without anosmia. He maintained normal morning erections, shaved daily, and had a testicular volume of approximately 10 cc.

F7, a 63-year-old male, exhibited profound self-observed anosmia, cryptorchidism, eunuchoid habitus, osteoporosis, Tanner stage 2, and a high-pitched voice. Laboratory assessments revealed extremely low testosterone levels of 140 ng/dL, along with suppressed gonadotropins (LH at 0.17 mIU/mL and FSH at 1.04 mIU/mL). Bone densitometry indicated significant osteopenia with a T-score of -2.8 in the spine.

F8, a 53-year-old male, reported experiencing occasional fatigue, absence of anosmia, gynecoid fat distribution, gynecomastia, and sporadic morning erections. Laboratory evaluations showed severely low testosterone levels at 190 ng/dL, with suppressed LH (0.1 mIU/mL) and FSH (0.31 mIU/mL).

Lastly, F9, a 58-year-old male, presented with no significant clinical features other than suppressed testosterone levels of 22.8 ng/dL, with no anosmia.

All affected male family members began testosterone replacement therapy at the time of diagnosis, meaning their age at diagnosis matched the age when treatment started in each case. For some individuals, particularly in the index case, diagnosis was significantly delayed because they did not seek medical attention due to minimal perceived symptoms.

After the index case was diagnosed with KS, targeted screening of family members identified additional affected relatives. However, long-term follow-up was limited in some cases.

The age of diagnosis, comorbidities, treatment, and testosterone levels of the affected family members are summarized in **Table 1**, detailing both pre-treatment and post-treatment conditions.

**Table 1:** Clinical features and treatment of the affected family members

Family member	Age	Sex	Known Comorbidities	Prescribed treatment	Testosterone levels (ng/dL)^1^
F1	65	F	Hypertension Dyslipidemia Ischemic CVA	No treatment required	Not available
F2	33	M	No known comorbidities	Testosterone enanthate: - 250 mg IM monthly	76 /1015
F3	27	M	Asthma	Testosterone enanthate: - 250 mg IM monthly	253 / Not available
F4	26	M	No known comorbidities	Testosterone enanthate: - 250 mg IM monthly	154 / Not available
F5	62	M	Dyslipidemia Sensorineural hypoacusis	Testosterone enanthate: - 250 mg IM monthly	140 / 720
F6	60	M	Obesity	Testosterone enanthate: - 250 mg IM monthly	Not available / 518
F7	63	M	CML Dyslipidemia Sensorineural hypoacusis	Testosterone enanthate: - 250 mg IM every 3 weeks	140 /3014^2^
F8	53	M	No known comorbidities	Testosterone enanthate: - 125 mg IM monthly	190 / Not available
F9	58	M	Scoliosis	Topical testosterone (AndroGel): - 50 mg daily	22.8 / 544

1 Testosterone levels: pre-treatment/post-treatment

2 Dosage later adjusted to 125 mg every 2 weeks; no further follow-up data available.

Additionally, to better illustrate the genetic findings within this family, the presence or absence of the identified mutations in *ANOS1* and *GNRHR* for each individual is listed in **Table 2**.

**Table 2:** Presence of *ANOS1* and *GNRHR* mutations in affected family members under study

Family member	*ANOS1* *(c.78_108del)*^1^	*GNRHR* *(c.974del)*
F1	✓	✓ (Het)
F2	✓	–
F3	✓	–
F4	✓	–
F5	✓	✓ (Het)
F6	–	✓ (Het)
F7	✓	–
F8	✓	✓ (Het)
F9	✓	–

✓ = Mutation present; – = Mutation absent.

Het = Heterozygous

1 Individual F1 is heterozygous for the *ANOS1* mutation, consistent with carrier status. All other affected males are hemizygous, as expected for an X-linked gene.

## DISCUSSION

Kallmann syndrome is a genetically heterogeneous condition that underscores the complex relationship between genotype and phenotype. As identified in the VarSome database, 22 genes are associated with KS. Among these, a key gene is *ANOS1*, formerly known as *KAL1*, which has been linked to 100 pathogenic variants and 94 variants of uncertain significance across various diseases. For *GNRHR* variants, there are 51 pathogenic variants and 44 variants of uncertain significance reported (^7^).

Although the VarSome database documents mutations such as *ANOS1* X-linked (c.78_108del, p.Pro27Serfs18) and *GNRHR* autosomal recessive (c.974del, p.Tyr325Phefs6), no pathogenic reports exist for these mutations (^7^). Therefore, to our knowledge, these mutations are novel and not only corroborate existing literature but also extend it.

The data collected on affected family members regarding clinical manifestations enable us to identify the inheritance pattern associated with the *ANOS1* mutation, as described in the literature. For instance, subject F1 exemplifies the asymptomatic carrier status phenomenon in females with X-linked recessive mutations (^1^). Additionally, this data allows us to observe the variable relationships between genetic factors and phenotypic expressions. Recent studies emphasized the oligogenic nature of KS, highlighting that mutations in multiple genes contribute to the observed clinical variability (^1^,^2^).

Evidence has indicated that mutations in the *ANOS1* gene are linked to significant clinical manifestations such as anosmia and hypogonadism (^1^,^2^). These manifestations are attributed to the gene’s critical role in axonal guidance and cell adhesion, which are essential for the migration of GnRH neurons and the development of the olfactory bulb (^1^,^2^). Mutations in the *ANOS1* gene are primarily associated with severe phenotypes, including anosmia and cryptorchidism (^3^). Nevertheless, the anosmin 1 protein is also expressed in various tissues, including the cerebellum, inner ear, kidneys, and skin. Hence, in addition to the typical features of KS, individuals with mutations in *ANOS1* have also been reported with defects such as renal agenesis, cleft palate, mirror movements, and sensorineural hearing loss (^5^,^6^).

Similarly, mutations in the *GNRHR* gene, which are inherited in an autosomal recessive manner, interfere with GnRH receptor signaling. This typically results in normosmic nCHH, where affected individuals maintain a normal sense of smell associated with low levels of gonadotropins (^3^).

In the reported cases, males with the *ANOS1* mutation exhibited several classic features of KS, including anosmia, delayed puberty, and cryptorchidism. These symptoms correlate with the gene’s role in neural adhesion and the migration of GnRH neurons. However, it is important to note that although mutations in *ANOS1* typically lead to significant phenotypic anomalies, three family members affected by this mutation only displayed mild symptoms of hypogonadism without self-observed or clinical manifestations of anosmia (F3, F8, and F9) (^2^,^8^).

Additionally, homozygous mutations in the *GNRHR* gene are associated with milder hypogonadism that occurs without anosmia, consistent with the clinical manifestation of F6 (^1^,^2^).

Furthermore, given the wide range of phenotypic manifestations associated with the *ANOS1* mutation found in the literature, it can be inferred that it may explain the sensorineural hearing loss of F5 (^6^,^9^). Nonetheless, there is insufficient data to definitively state that this symptom is directly caused by his genetic mutation.

The phenotypic variability observed in this family underscores the impact of oligogenic inheritance. Specifically, digenic combinations of mutations in the *ANOS1* and *GNRHR* genes were linked to more severe phenotypes in the affected males, including compounded hypogonadism and anosmia. This finding supports the hypothesis that interactions between multiple genetic loci influence clinical outcomes, as described elsewhere (^3^). Additionally, the coexistence of mutations that exacerbate clinical severity corroborates studies suggesting additive effects in digenic and oligogenic cases (^2^). However, the primary role of the *ANOS1* gene is also evident, as mutations in this gene alone can still lead to clinical manifestations of KS, although in some instances, the phenotypic presentation may not be as pronounced (^2^,^8^).

## LIMITATIONS AND FUTURE DIRECTIONS

This study underscores significant genotype-phenotype correlations but does not include functional studies to confirm the pathogenicity of the identified mutations. Additionally, delayed diagnosis in some individuals and limited long-term follow-up of affected family members constitute important limitations of this report. Future research should aim to validate these findings through *in vitro* or *in vivo* models to understand the molecular mechanisms underlying these mutations. Moreover, expanding genetic studies to larger KS cohorts may elucidate additional oligogenic interactions, providing deeper insights into the disease’s pathophysiology.

In conclusion, this familial case study adds to the growing body of evidence supporting the role of oligogenic inheritance in Kallmann syndrome. The identification of novel mutations in *ANOS1* and *GNRHR* reinforces the genetic complexity of the disorder and underscores the critical role of gene analysis in advancing diagnostic and therapeutic strategies. From a clinical perspective, these findings highlight the necessity of comprehensive genetic evaluation in Kallmann syndrome, particularly in familial cases. Genetic testing facilitates the identification of novel and compound mutations, enabling tailored therapeutic strategies. Studies advocate for the use of next-generation sequencing to uncover oligogenic contributions in KS, which could elucidate unexplained cases and guide genetic counseling.

## Data Availability

datasets related to this article will be available upon request to the corresponding author.
